# Supplementary material for: Zebrafish Avatar-test forecasts clinical response to chemotherapy in patients with colorectal cancer
Source: Nat Commun. 2024 Jun 5;15:4771. doi: 10.1038/s41467-024-49051-0 (PMC11153622; doi:10.1038/s41467-024-49051-0)
Supplement: Supplementary file 1 — Supplementary Information [file 41467_2024_49051_MOESM1_ESM.pdf]

## Supplementary Figure 1

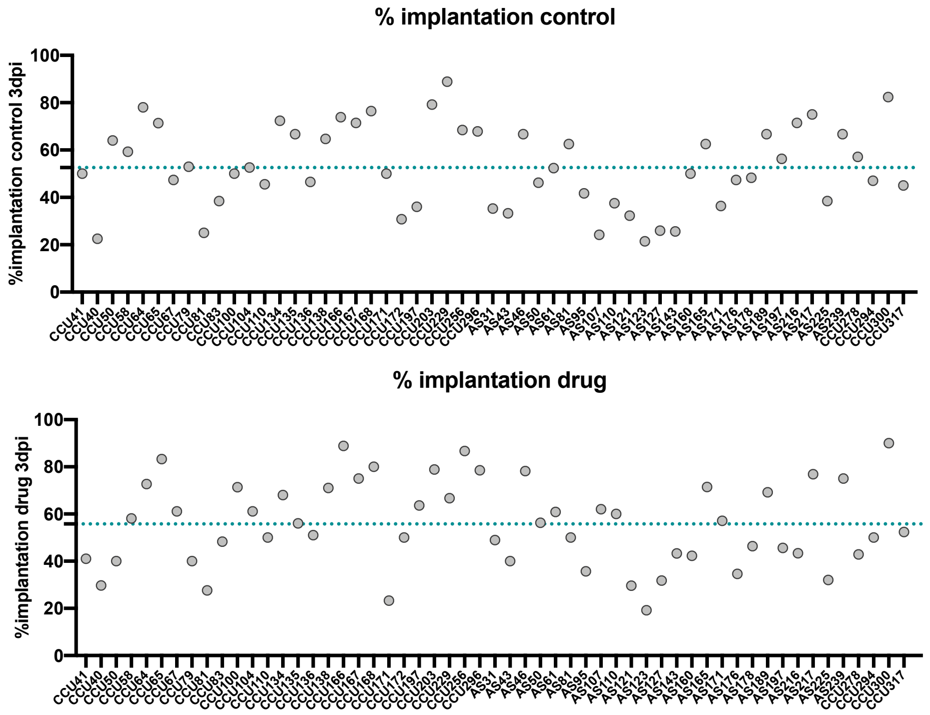


**Supplementary Figure 1. Implantation rates of CRC zAvatars.** Percentage of implantation, defined as the number of zAvatars with tumor divided by the total number of zAvatars at 3dpi, is shown for each zAvatar. Source data are provided as a Source Data file.

# Supplementary Figure 2

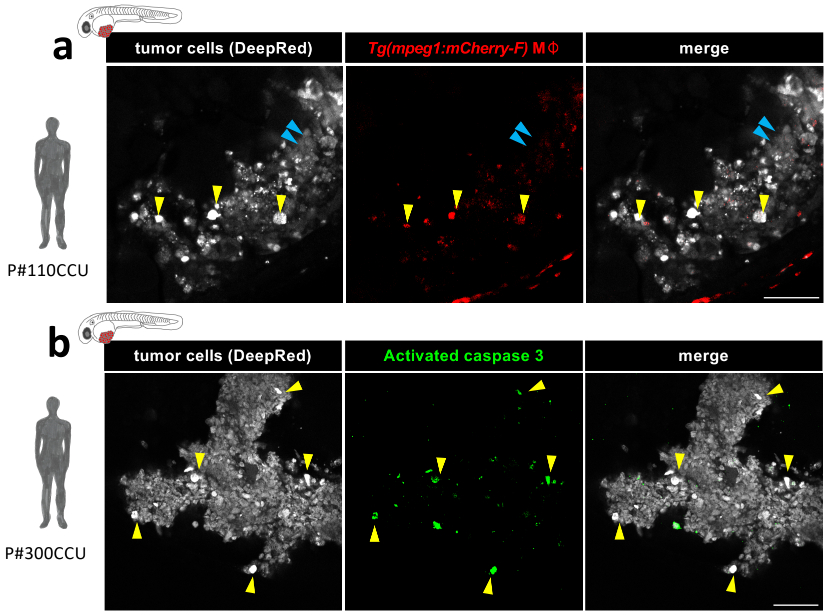


**Supplementary Figure 2:** (a) Example of tumor cells injected into the zebrafish transgenic line *Tg(mpeg1:mCherry-F)*, a zebrafish macrophage reporter line. Yellow arrows indicate the brightest cells, likely undergoing phagocytosis. Blue arrows highlight cells with less intense staining. Host macrophages surrounding the tumor can be observed at the bottom of the image. (b) Example of tumor cells stained with activated caspase 3 (in green). Scale bars represent 50 µm. Yellow arrows indicate the brightest cells, which coincide with cells undergoing apoptosis.

# Supplementary Figure 3

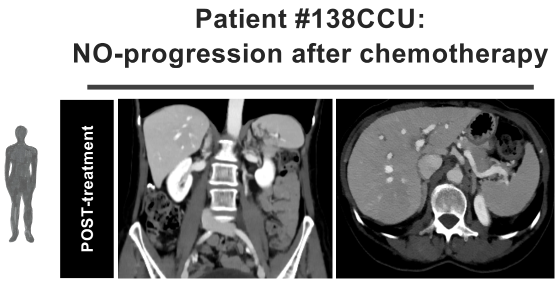


**Supplementary Figure 3:** Post-operative follow-up imaging of P#138CCU revealed no signs of disease recurrence, representing an example of a patient with no-progression. The image of the liver section confirms the abscence of recurrence.

## Supplementary Figure 4

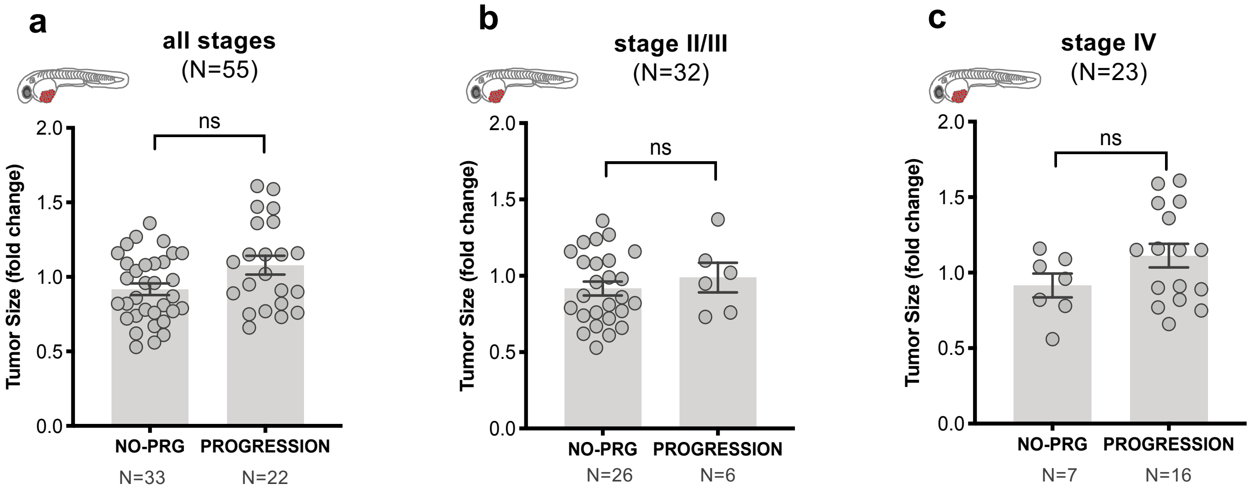


**Supplementary Figure 4. Tumor size is not predictive of patient clinical response to treatment.** (a) Fold change of tumor size (number of tumor cells) in zAvatars from patients with no-progression (N=33 patients, a total of 667 zAvatars analyzed) is not different from those experiencing progression (N=22 patients, a total of 518 zAvatars analyzed); total N=55 patients, p=0.0702. (b) The same trend was observed in stage II/III patients: N=26 patients with no-progression (530 zAvatars analyzed) vs N=6 patients with progression (137 zAvatars analysed); total of N=32 patients, p=0.5797. (c) Same as (a) but considering samples from stage IV patients: N=7 patients with no-progression (137 zAvatars analized) vs N=16 patients with progression (381 zAvatars analyzed; total N=23 patients, p=0.2413. Results are expressed as AVG±SEM. N=number of patients. Data were analyzed using unpaired two-sided Mann–Whitney test: (ns) > 0.05.

# Supplementary Figure 5

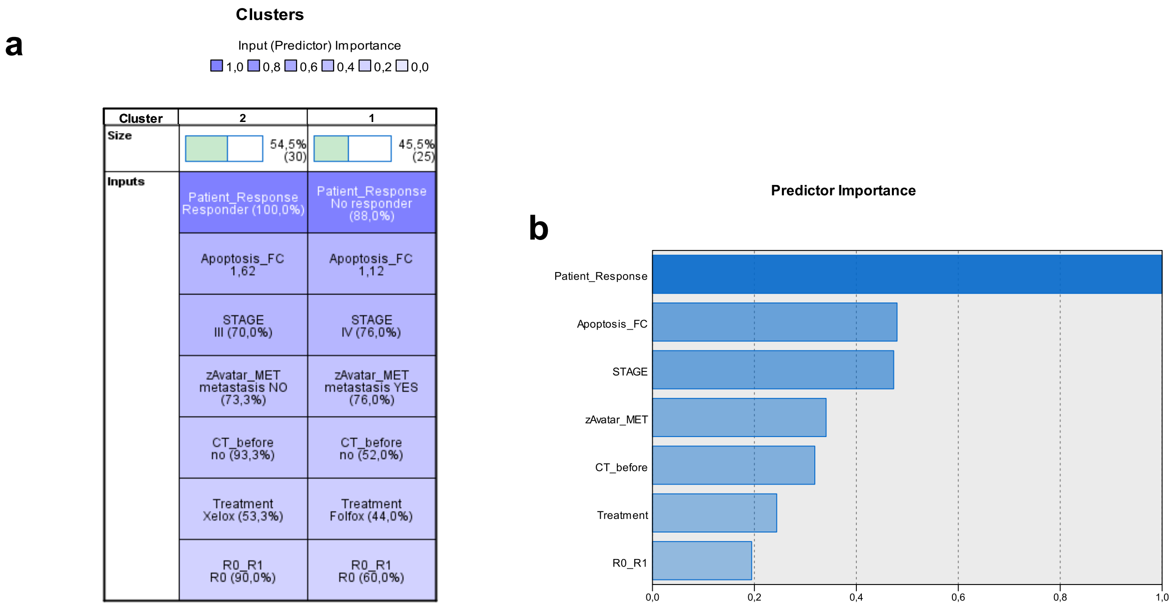


**Supplementary Figure 5. Association of exploratory variables and patient clinical response to treatment.** (a) A multivariate classification "Two-step cluster" revealed two groups of patients with different characteristics. The variables included were those that showed statistical significance in the bivariate non-parametric analysis presented in Supplementary Table 3. (b) Relative predictor importance of each variable according to the multivariate cluster outcome. "Apoptosis FC", "Tumor Stage" and "zAvatar metastasis" were the most important variables to differentiate "responders" *vs* "non-responders". Source data are provided as a Source Data file.

# Supplementary Figure 6

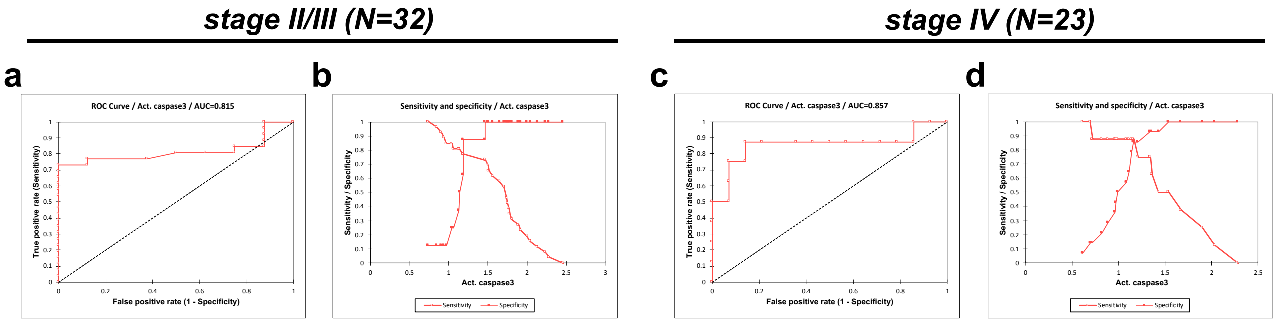


**Supplementary Figure 6:** (a) ROC analysis of the average fold change of apoptosis in stage II/III patients (N=32). (b) A cut-off value of 1.47 was identified as the optimal threshold. (c) ROC analysis of the average fold change of apoptosis in stage IV patients (N=23). (d) A cut-off value of 1.18 was identified as the optimal threshold. Source data are provided as a Source Data file.

**Supplementary Table 1:** Characteristics of CRC patients included in the study.

| Characteristic | N=55 |
|---|---|
| **Median age (years, range)** | 64(44-82) |
| **Sex, n (%)** | |
| Male | 18 (32,7) |
| Female | 37 (67,3) |
| **Sample type, n (%)** | |
| Colon | 28 (50,9) |
| Right | 16 (29,1) |
| Left | 4 (7,3) |
| Sigmoid | 8 (14,5) |
| Rectum | 7 (12,7) |
| Liver metastasis | 20 (36,4) |
| **Stage, n (%)** | |
| II | 5 (9,1) |
| III | 27 (49,1) |
| IV | 23 (41,8) |
| **Tumor subtype, n (%)** | |
| conventional | 45 (81,8) |
| mucinous | 9 (16,4) |
| micropapillary | 1 (1,8) |
| **KRAS, n (%)** | |
| wild-type | 16 (29,1) |
| mutated | 17 (30,9) |
| unknown | 22 (40) |
| **BRAF, n (%)** | |
| wild-type | 13 (23,6) |
| mutated | 5 (9,1) |
| unknown | 37 (67,3) |
| **Grade, n (%)** | |
| G1 | 15 (27,3) |
| G2 | 30 (54,5) |
| G3 | 8 (14,5) |
| unknown | 2 (3,6) |
| **Intratumoral lymphocytes, n (%)** | |
| absent | 19 (34,5) |
| present | 36 (65,5) |
| **Microsatellite status, n (%)** | |
| MSS | 39 (70,9) |
| MSI | 4 (7,3) |
| unknown | 12 (21,8) |
| **Residual tumor classification, n (%)** | |
| R0 | 42 (76,4) |
| R1 | 13 (23,6) |
| **Perineural invasion, n (%)** | |
| yes | 16 (29,1) |
| no | 26 (47,3) |
| unknown | 13 (23,6) |
| **Chemotherapy before surgery, n (%)** | |
| yes | 14 (25,5) |
| no | 41 (74,5) |
| **Patient status 12months follow-up, n (%)** | |
| stable | 33 (60) |
| progression | 22 (40) |

Frequencies and percentages of demographic variables of patients (N=55). Source data are provided as a Source Data file.

**Supplementary Table 2:** CRC chemotherapy regimens and final concentration of drugs diluted in E3 medium.

| **Chemotherapy regimen** | **Drugs (final concentration diluted in E3)** |
|---|---|
| **FOLFOX** | 5-FU (4260μM) + Folinic acid (185μM) + Oxaliplatin (81μM) |
| **FOLFIRI** | 5-FU (4260μM) + Folinic acid (185μM) + Irinotecan (80μM) |
| **CAPOX** | 5-FU (4260μM) + Oxaliplatin (81μM) |
| **CAPIRI** | 5-FU (4260μM) + Irinotecan (80μM) |
| **FUFOL** | 5-FU (4260μM) + Folinic acid (185μM) |
| **Bevacizumab** | 250μg/mL |
| **Cetuximab** | 100μg/mL |

**Supplementary Table 3:** Bivariate non-parametric analysis of demographic, clinical and zAvatar variables, considering "Patient Response" as the dependent variable.

| Association of clinical and zAvatar variables and "Patient Response" | | |
|---|---|---|
| **Clinical variables** | | **P** |
| **Cancer Staging** | II, III, IV | **<0.001** |
| **Chemotherapy before surgery** | Yes/No | **0.005** |
| **Chemotherapy after surgery** | FOLFOX, FOLFIRI, etc | **0.011** |
| **Residual (R) tumor classification** | R0/R1 | **0.014** |
| **Type of sample** | Right colon, left colon, etc | **0.047** |
| BRAF | Wild-type, mutated, unknown | 0.06 |
| Age | (44-82) | 0.12 |
| N (node) staging | N0, N1, N2 | 0.142 |
| KRAS | Wild-type, mutated, unknown | 0.221 |
| Tumor grade | G1, G2, G3 | 0.259 |
| Gender | Male/Female | 0.291 |
| Tumor subtype | Conventional, mucinous, etc | 0.329 |
| Intratumoral lymphocytes | Present/Absent | 0.354 |
| Perineural invasion | Present/Absent | 0.394 |
| Microsatellite status | MSI/MSS | 0.735 |
| **zAvatar variables** | | |
| **Apoptosis FC** | % of activated caspase3 in control *vs* treatment | **<0.001** |
| **MET (presence of metastases)** | Presence/Absence of micrometastases | **0.004** |
| Tumor Size FC | Nº of tumor cells in control *vs* treatment | 0.071 |
| Implantation FC | % of implantation in control *vs* treatment | 0.4244 |
| Mito FC | % of human mitochondria in control *vs* treatment | 0.531 |

Categorical variables were compared using a two-sided chi-square test, with statistical significance indicated by bold formatting. Source data are provided as a Source Data file.
