# Supplementary material for: Zebrafish Avatar-test forecasts clinical response to chemotherapy in patients with colorectal cancer
Source: Nat Commun. 2024 Jun 5;15:4771. doi: 10.1038/s41467-024-49051-0 (PMC11153622; doi:10.1038/s41467-024-49051-0)
Supplement: Supplementary file 3 — Description of Additional Supplementary Files [file 41467_2024_49051_MOESM3_ESM.pdf]

Description of Additional Supplementary Files

Supplementary Data 1: Clinical data of CRC patients included in the study.

Supplementary Data 2: Patient and zAvatars outcomes and correlations.

Supplementary Movie 1: Time-lapse movie of a liver metastasis sample from a colon cancer patient injected into Tg(*mpeg1:mCherry-F)*, a zebrafish macrophage reporter line. The video specifically focuses on the tail region.
