# Supplementary material for: Zebrafish Avatar-test forecasts clinical response to chemotherapy in patients with colorectal cancer
Source: Nat Commun. 2024 Jun 5;15:4771. doi: 10.1038/s41467-024-49051-0 (PMC11153622; doi:10.1038/s41467-024-49051-0)
Supplement: Supplementary file 7 — Reporting Summary [file 41467_2024_49051_MOESM7_ESM.pdf]

nature portfolio

Corresponding author(s): Rita Fior

Last updated by author(s): May 8, 2024

Reporting Summary

Nature Portfolio wishes to improve the reproducibility of the work that we publish. This form provides structure for consistency and transparency in reporting. For further information on Nature Portfolio policies, see our Editorial Policies and the Editorial Policy Checklist.

Statistics

For all statistical analyses, confirm that the following items are present in the figure legend, table legend, main text, or Methods section.

n/a	Confirmed	
☐	☒	The exact sample size (*n*) for each experimental group/condition, given as a discrete number and unit of measurement
☐	☒	A statement on whether measurements were taken from distinct samples or whether the same sample was measured repeatedly
☐	☒	The statistical test(s) used AND whether they are one- or two-sided<br>*Only common tests should be described solely by name; describe more complex techniques in the Methods section.*
☐	☒	A description of all covariates tested
☐	☒	A description of any assumptions or corrections, such as tests of normality and adjustment for multiple comparisons
☐	☒	A full description of the statistical parameters including central tendency (e.g. means) or other basic estimates (e.g. regression coefficient) AND variation (e.g. standard deviation) or associated estimates of uncertainty (e.g. confidence intervals)
☐	☒	For null hypothesis testing, the test statistic (e.g. *F*, *t*, *r*) with confidence intervals, effect sizes, degrees of freedom and *P* value noted<br>*Give P values as exact values whenever suitable.*
☒	☐	For Bayesian analysis, information on the choice of priors and Markov chain Monte Carlo settings
☒	☐	For hierarchical and complex designs, identification of the appropriate level for tests and full reporting of outcomes
☒	☐	Estimates of effect sizes (e.g. Cohen's *d*, Pearson's *r*), indicating how they were calculated

Our web collection on statistics for biologists contains articles on many of the points above.

Software and code

Policy information about availability of computer code

Data collection	Zeiss Zen software (Zen blue edition) from Zeiss LSM 980, and Fusion software (version 1.1.1.17) from BC43 Andor confocal microscopes were used. Clinical data from patients was collected from their respective medical electronic records by the attending physician: Siemens Soarian Clinicals (Hospital Prof. Doutor Fernando Fonseca, Amadora, Portugal) and Citrix Systems (Champalimaud Clinical Centre, Lisbon, Portugal).
Data analysis	Data analysis was performed using FIJI/Image J version 2.0.0-rc-30), GraphPad Prism (version 8.2.1, 279), IBM SPSS (version 28) and XLStat (version 2022.4.1) softwares. Images were analyzed using ImageJ software, using the Cell Counter plugin. The tumor size (number of tumor cells), percentage of activated caspase 3 and percentage of micrometastasis were quantified manually, counting all cells in every slice of the tumor (from Zfirst to Zlast).<br>Receiver operating characteristic (ROC) analysis was performed using XLStat software, considering response to treatment (no-progression disease) as a positive event.<br>Kaplan–Meier curves were performed using GraphPad Prism software and compared with the log-rank test.<br>A multivariate classification analysis was computed using the “Two-Step Clusters” and “Classification and Regression Trees” (CRT) algorithm with IBM SPSS Statistics version 28. The independent variables were selected according to the significant association with “Patient Response”, in the bivariate analysis context. Using the CRT growing method with non-cross-validation, a five minimum cases in parent and child nodes with tree depth of two were specified.

For manuscripts utilizing custom algorithms or software that are central to the research but not yet described in published literature, software must be made available to editors and reviewers. We strongly encourage code deposition in a community repository (e.g. GitHub). See the Nature Portfolio guidelines for submitting code & software for further information.

Data

Policy information about availability of data

All manuscripts must include a data availability statement. This statement should provide the following information, where applicable:

- Accession codes, unique identifiers, or web links for publicly available datasets
- A description of any restrictions on data availability
- For clinical datasets or third party data, please ensure that the statement adheres to our policy

All data in this study are available in the manuscript or the Supplementary materials. Other data related to this work are available from the corresponding author upon request. Source data are provided with this paper.

Research involving human participants, their data, or biological material

Policy information about studies with human participants or human data. See also policy information about sex, gender (identity/presentation), and sexual orientation and race, ethnicity and racism.

Reporting on sex and gender	Patients were recruited irrespective of their sex, resulting in a study population that includes both male (n=18) and female (n=37) participants. Sex and gender were not factors considered as factors in the study design. The variable collected by the clinical registration software of both hospitals specifically pertains to sex, not gender.<br>This data was collected only for the purpose of characterizing population demographics.
Reporting on race, ethnicity, or other socially relevant groupings	In our study, data on race, ethnicity, or other social relevant groupings were not collected. Based on the study's objectives, these factors were not included in the study design or data collection process.
Population characteristics	The covariate-relevant population characteristics of the human research participants in our study include a range of demographic, clinical, surgical, and pathological data: date of birth, sex, KRAS and BRAF status, tumor type, tumor grade and tumor subtype, microsatellite status, intratumoral infiltrating lymphocytes, pathological staging, residual tumor classification, and perineural invasion. Regarding treatment history: chemotherapy and/or radiotherapy before surgery (when applied), type of chemotherapy after surgery, number of cycles and duration. The outcomes measures included the assessment of progression or no-progression disease and progression-free survival in the 12 months after initiation of chemotherapy (see Supplementary Table 1 for details).
Recruitment	The study was explained to eligible patients at Champalimaud Clinical Centre and Hospital Prof. Doutor Fernando Fonseca, typically during the consultation prior to surgery.<br>These patients are followed by the physicians who are part of the study team, so the approach to the potential participants and recruitment was conducted by their respective physicians, as well as the obtention of the signed informed consent.
Ethics oversight	The study was approved by the Ethics Committees of the Champalimaud Foundation and Hospital Prof. Doutor Fernando Fonseca, and was conducted in accordance with ethical principles founded in the Declaration of Helsinki. All patients signed an informed consent to participate in the study.

Note that full information on the approval of the study protocol must also be provided in the manuscript.

Field-specific reporting

Please select the one below that is the best fit for your research. If you are not sure, read the appropriate sections before making your selection.

☒ Life sciences ☐ Behavioural & social sciences ☐ Ecological, evolutionary & environmental sciences

For a reference copy of the document with all sections, see nature.com/documents/nr-reporting-summary-flat.pdf

Life sciences study design

All studies must disclose on these points even when the disclosure is negative.

Sample size	The sample-size calculation was conducted referencing findings from our prior study by Fior et al (PNAS 2017, https://doi.org/10.1073/pnas.1618389114). Given the established parameters of 85% sensitivity and 85% specificity, coupled with a significance level of 95% and a precision of 90%, our analysis indicates that a sample size of 49 patients diagnosed with CRC, and who have provided results in the zAvatar, is required to ensure robust statistical power and meaningful outcomes (Arifin WN. Sample size calculator (web) 2023, available from:http://wnarifin.github.io).<br><br>For the quantification of implantation rates, tumor size, % of apoptosis, and % of micrometastasis, confocal images acquired with the Zeiss LSM 980 were individually and manually quantified. As these are human samples, the quantity of cells obtained for each experiment and the number of zebrafish embryos analyzed in the end are variable. This variability depends not only on the initial sample size but also on intrinsic characteristics of the tumor, such as cellularity.
Data exclusions	To enable statistical analysis, we established a minimum requirement of four zebrafish embryos analyzed for each experimental condition. Thus, patients whose zebrafish avatar reported low engraftment or induced host death (n<4 embryos for each experimental condition) were

excluded from the analysis. This exclusion criteria was applied to four experiments: zAvatars from P#83AS (n=2 in control and n=5 in drug); P#170CCU (n=2 in control and n=4 in drug); P#211CCU (n=3 in control and n=3 in drug); P#225CCU (n=7 in control and n=3 in drug).

Replication

The majority of experiments were performed only once for each patient due to the limited amount of human sample available. We do not expand the patient tumor sample in culture to avoid caveats of selection. Thus, in the majority of cases, each human specimen was processed, and all tumor cells were used for the generation of zAvatars and subsequent therapy testing. The main limiting factors affecting the reproducibility of these experiments are firstly the quantity of tumor cells obtained from each specimen, and the occurrence of zAvatars that either died or developed cardiac edema during the experiment.

Nevertheless, for certain patient’s samples, it was possible to cryopreserve more than one vial and indeed we were able to repeat experiments and obtained very similar results. This was observed in the cases of zAvatar #61AS, #110AS, #136CCU and #139CCU demonstrating the reproducibility of our experimental design.

Despite the study’s unique limitations, efforts were made to standardize procedures, including the human sample processing protocol, microinjection technique and quantifications performed, in order to minimize variability and ensure consistency in the results. In this regard, we have already published two methods papers on zebrafish xenografts generation (Martinez-Lopez et al, JoVE 2021; DOI: 10.3791/62373-v ; Costa et al, Current Protocols 2022, https://doi.org/10.1002/cpz1.415).

Randomization

The patients were not randomized. All patients received treatment according to the standard of care. Concurrently, the same treatment was tested on each patient's zAvatar. In cases where there was sufficient sample material, additional treatments were also evaluated.
Finally, several covariates of each patient (Supplementary Data File 1) were taken into consideration for assessing “patient response to treatment”, by conducting a multivariate regression analysis.

Blinding

The zAvatar response to treatment was blindly compared with the patient's clinical response 12 months after initiating chemotherapy. We categorized each patient's zAvatar as either sensitive or resistant. Subsequently, this information was cross-referenced with the oncologist's assessment, which defined each patient as having either progression or no-progression disease (stable) after treatment.

Reporting for specific materials, systems and methods

We require information from authors about some types of materials, experimental systems and methods used in many studies. Here, indicate whether each material, system or method listed is relevant to your study. If you are not sure if a list item applies to your research, read the appropriate section before selecting a response.

Materials & experimental systems

n/a	Involved in the study
☐	☒ Antibodies
☐	☐ Eukaryotic cell lines
☐	☐ Palaeontology and archaeology
☐	☒ Animals and other organisms
☐	☒ Clinical data
☐	☐ Dual use research of concern
☐	☐ Plants

Methods

n/a	Involved in the study
☐	☐ ChIP-seq
☐	☐ Flow cytometry
☐	☐ MRI-based neuroimaging

Antibodies

Antibodies used

Primary antibodies: anti-Cleaved Caspase3 (Asp175) (rabbit, Cell signaling, 1:100, cat#9661), anti-Human mitochondria (mouse, Merck Millipore, 1:100, cat#MAB1273). Secondary antibodies:anti-mouse DyLight 488 (Thermo Fisher Scientific, 1:400, cat#10688674), anti-rabbit DyLight 650 (Thermo Fisher Scientific, 1:400, cat#84546), anti-rabbit DyLight 594 (Thermo Fisher Scientific, 1:400, cat#10108403).

Validation

Cleaved Caspase-3 (Asp175) antibody detects endogenous levels of the large fragment (17/19 kDa) of activated caspase-3 resulting from cleavage adjacent to Asp175. This antibody does not recognize full length caspase-3 or other cleaved caspases (see Cell Signaling website).

Anti-Human mitochondria antibody gives mitochondrial staining on all human cell types and does not cross react with rat and mouse tissue. It is routinely evaluated by immunohistochemistry on heart ventricle cells (see Merck website).

Eukaryotic cell lines

Policy information about cell lines and Sex and Gender in Research

Cell line source(s)

State the source of each cell line used and the sex of all primary cell lines and cells derived from human participants or vertebrate models.

Authentication	*Describe the authentication procedures for each cell line used OR declare that none of the cell lines used were authenticated.*
Mycoplasma contamination	*Confirm that all cell lines tested negative for mycoplasma contamination OR describe the results of the testing for mycoplasma contamination OR declare that the cell lines were not tested for mycoplasma contamination.*
Commonly misidentified lines (See ICLAC register)	*Name any commonly misidentified cell lines used in the study and provide a rationale for their use.*

Palaeontology and Archaeology

Specimen provenance	*Provide provenance information for specimens and describe permits that were obtained for the work (including the name of the issuing authority, the date of issue, and any identifying information). Permits should encompass collection and, where applicable, export.*
Specimen deposition	*Indicate where the specimens have been deposited to permit free access by other researchers.*
Dating methods	*If new dates are provided, describe how they were obtained (e.g. collection, storage, sample pretreatment and measurement), where they were obtained (i.e. lab name), the calibration program and the protocol for quality assurance OR state that no new dates are provided.*

☐ Tick this box to confirm that the raw and calibrated dates are available in the paper or in Supplementary Information.

Ethics oversight	*Identify the organization(s) that approved or provided guidance on the study protocol, OR state that no ethical approval or guidance was required and explain why not.*

Note that full information on the approval of the study protocol must also be provided in the manuscript.

Animals and other research organisms

Policy information about studies involving animals; ARRIVE guidelines recommended for reporting animal research, and Sex and Gender in Research

Laboratory animals	In vivo experiments were performed in zebrafish model (Danio rerio), which was maintained and handled in accordance with European Animal Welfare Legislation, Directive 2010/63/EU and Champalimaud Fish Platform Program.<br>Adult zebrafish, both males and females, were housed in 3.5L tanks with a maximum population of 30 fish per tank, in a running water system, fed twice a day and maintained in a temperature and humidity controlled environment. They also followed a day-night automatic cycle of 14 hours of light plus 10 hours of dark.<br>Adults were used to breed, and the experiments were performed in 2 days post-fertilization (dpf) zebrafish embryos.<br>For this study, we used a combination of four different backgrounds: wild-type mutants (Nacre or Casper); a genetically modified zebrafish line, Tg(Fli1:eGFP), for visualization of blood and lymphatic vessels; and Tg(mpeg1:mCherry-F), a zebrafish macrophage reporter line.
Wild animals	The study did not involve wild animals.
Reporting on sex	We used zebrafish embryos in which gender is not yet determined (from 2dpf until 5dpf).
Field-collected samples	The study did not involve field-collected samples.
Ethics oversight	The Portuguese institutional organizations- ORBEA (Órgão de Bem-Estar e Ética Animal / Animal Welfare and Ethics Body) and DGAV (Direção Geral de Alimentação e Veterinária / Directorate General for Food and Veterinary) have approved this study and its corresponding protocols.

Note that full information on the approval of the study protocol must also be provided in the manuscript.

Clinical data

Policy information about clinical studies

All manuscripts should comply with the ICMJEguidelines for publication of clinical research and a completedCONSORT checklist must be included with all submissions.

Clinical trial registration	We are in the process of registration; this study is part of an umbrella observational study that includes other types of cancer.
Study protocol	We are in the process of registration; the study protocol will be available upon registration. However, upon request, we can also provide the approved study protocol.
Data collection	Clinical data from patients was collected from their respective medical electronic records by the attending physician: Siemens Soarian Clinicals (Hospital Prof. Doutor Fernando Fonseca, Amadora, Portugal) and Citrix Systems (Champalimaud Clinical Centre, Lisbon, Portugal). At the time of zAvatars generation the only information required was the type of chemotherapy that the patient underwent after surgery. The remaining variables were collected at the end of the study, following the assessment of patient response (12 months after treatment).

Outcomes	The follow-up of CRC patients was performed according to ESMO and NCCN guidelines. Classification of progression/no-progression disease was based in imagiological findings (CT, MRI, PET), clinical assessment, histological confirmation, all discussed in multidisciplinary team (MDT) meetings.<br>In stage II/III patients, a clinical response to treatment was classified as "no-progression" if there was no evidence of disease recurrence within 12 months after treatment initiation. On the other hand, progression was defined as recurrence of the disease either at the same site as the primary tumor (local recurrence) or in a distant location (distant recurrence/metastasis), i.e., emergence of new imagiological findings in situ or at distance.<br>In stage IV patients, a clinical response to treatment was defined as "no-progression" when there was no increase in the remaining disease or evidence of de novo disease (in other words, stable disease). Conversely, progression was defined as an increase of the previous lesions or appearance of new disease during the follow-up period.

Dual use research of concern

Policy information about dual use research of concern

Hazards

Could the accidental, deliberate or reckless misuse of agents or technologies generated in the work, or the application of information presented in the manuscript, pose a threat to:

No	Yes	
☒	☐	Public health
☒	☐	National security
☒	☐	Crops and/or livestock
☒	☐	Ecosystems
☒	☐	Any other significant area

Experiments of concern

Does the work involve any of these experiments of concern:

No	Yes	
☒	☐	Demonstrate how to render a vaccine ineffective
☒	☐	Confer resistance to therapeutically useful antibiotics or antiviral agents
☒	☐	Enhance the virulence of a pathogen or render a nonpathogen virulent
☒	☐	Increase transmissibility of a pathogen
☒	☐	Alter the host range of a pathogen
☒	☐	Enable evasion of diagnostic/detection modalities
☒	☐	Enable the weaponization of a biological agent or toxin
☒	☐	Any other potentially harmful combination of experiments and agents

Plants

Seed stocks	*Report on the source of all seed stocks or other plant material used. If applicable, state the seed stock centre and catalogue number. If plant specimens were collected from the field, describe the collection location, date and sampling procedures.*
Novel plant genotypes	*Describe the methods by which all novel plant genotypes were produced. This includes those generated by transgenic approaches, gene editing, chemical/radiation-based mutagenesis and hybridization. For transgenic lines, describe the transformation method, the number of independent lines analyzed and the generation upon which experiments were performed. For gene-edited lines, describe the editor used, the endogenous sequence targeted for editing, the targeting guide RNA sequence (if applicable) and how the editor was applied.*
Authentication	*Describe any authentication procedures for each seed stock used or novel genotype generated. Describe any experiments used to assess the effect of a mutation and, where applicable, how potential secondary effects (e.g. second site T-DNA insertions, mosiacism, off-target gene editing) were examined.*

ChIP-seq

Data deposition

☐ Confirm that both raw and final processed data have been deposited in a public database such as GEO.

☐ Confirm that you have deposited or provided access to graph files (e.g. BED files) for the called peaks.

Data access links<br>*May remain private before publication.*	*For "Initial submission" or "Revised version" documents, provide reviewer access links. For your "Final submission" document, provide a link to the deposited data.*
Files in database submission	*Provide a list of all files available in the database submission.*
Genome browser session<br>(e.g. UCSC)	*Provide a link to an anonymized genome browser session for "Initial submission" and "Revised version" documents only, to enable peer review. Write "no longer applicable" for "Final submission" documents.*

Methodology

Replicates	*Describe the experimental replicates, specifying number, type and replicate agreement.*
Sequencing depth	*Describe the sequencing depth for each experiment, providing the total number of reads, uniquely mapped reads, length of reads and whether they were paired- or single-end.*
Antibodies	*Describe the antibodies used for the ChIP-seq experiments; as applicable, provide supplier name, catalog number, clone name, and lot number.*
Peak calling parameters	*Specify the command line program and parameters used for read mapping and peak calling, including the ChIP, control and index files used.*
Data quality	*Describe the methods used to ensure data quality in full detail, including how many peaks are at FDR 5% and above 5-fold enrichment.*
Software	*Describe the software used to collect and analyze the ChIP-seq data. For custom code that has been deposited into a community repository, provide accession details.*

Flow Cytometry

Plots

Confirm that:

- ☐ The axis labels state the marker and fluorochrome used (e.g. CD4-FITC).
- ☐ The axis scales are clearly visible. Include numbers along axes only for bottom left plot of group (a 'group' is an analysis of identical markers).
- ☐ All plots are contour plots with outliers or pseudocolor plots.
- ☐ A numerical value for number of cells or percentage (with statistics) is provided.

Methodology

Sample preparation	*Describe the sample preparation, detailing the biological source of the cells and any tissue processing steps used.*
Instrument	*Identify the instrument used for data collection, specifying make and model number.*
Software	*Describe the software used to collect and analyze the flow cytometry data. For custom code that has been deposited into a community repository, provide accession details.*
Cell population abundance	*Describe the abundance of the relevant cell populations within post-sort fractions, providing details on the purity of the samples and how it was determined.*
Gating strategy	*Describe the gating strategy used for all relevant experiments, specifying the preliminary FSC/SSC gates of the starting cell population, indicating where boundaries between "positive" and "negative" staining cell populations are defined.*

☐ Tick this box to confirm that a figure exemplifying the gating strategy is provided in the Supplementary Information.

Magnetic resonance imaging

Experimental design

Design type	*Indicate task or resting state; event-related or block design.*
Design specifications	*Specify the number of blocks, trials or experimental units per session and/or subject, and specify the length of each trial or block (if trials are blocked) and interval between trials.*
Behavioral performance measures	*State number and/or type of variables recorded (e.g. correct button press, response time) and what statistics were used to establish that the subjects were performing the task as expected (e.g. mean, range, and/or standard deviation across subjects).*

Acquisition

Imaging type(s) — Specify: functional, structural, diffusion, perfusion.

Field strength — Specify in Tesla

Sequence & imaging parameters — Specify the pulse sequence type (gradient echo, spin echo, etc.), imaging type (EPI, spiral, etc.), field of view, matrix size, slice thickness, orientation and TE/TR/flip angle.

Area of acquisition — State whether a whole brain scan was used OR define the area of acquisition, describing how the region was determined.

Diffusion MRI ☐ Used ☐ Not used

Preprocessing

Preprocessing software — Provide detail on software version and revision number and on specific parameters (model/functions, brain extraction, segmentation, smoothing kernel size, etc.).

Normalization — If data were normalized/standardized, describe the approach(es): specify linear or non-linear and define image types used for transformation OR indicate that data were not normalized and explain rationale for lack of normalization.

Normalization template — Describe the template used for normalization/transformation, specifying subject space or group standardized space (e.g. original Talairach, MNI305, ICBM152) OR indicate that the data were not normalized.

Noise and artifact removal — Describe your procedure(s) for artifact and structured noise removal, specifying motion parameters, tissue signals and physiological signals (heart rate, respiration).

Volume censoring — Define your software and/or method and criteria for volume censoring, and state the extent of such censoring.

Statistical modeling & inference

Model type and settings — Specify type (mass univariate, multivariate, RSA, predictive, etc.) and describe essential details of the model at the first and second levels (e.g. fixed, random or mixed effects; drift or auto-correlation).

Effect(s) tested — Define precise effect in terms of the task or stimulus conditions instead of psychological concepts and indicate whether ANOVA or factorial designs were used.

Specify type of analysis: ☐ Whole brain ☐ ROI-based ☐ Both

Statistic type for inference — Specify voxel-wise or cluster-wise and report all relevant parameters for cluster-wise methods.

(See Eklund et al. 2016)

Correction — Describe the type of correction and how it is obtained for multiple comparisons (e.g. FWE, FDR, permutation or Monte Carlo).

Models & analysis

n/a	Involved in the study
☐	☐ Functional and/or effective connectivity
☐	☐ Graph analysis
☐	☐ Multivariate modeling or predictive analysis

Functional and/or effective connectivity — Report the measures of dependence used and the model details (e.g. Pearson correlation, partial correlation, mutual information).

Graph analysis — Report the dependent variable and connectivity measure, specifying weighted graph or binarized graph, subject- or group-level, and the global and/or node summaries used (e.g. clustering coefficient, efficiency, etc.).

Multivariate modeling and predictive analysis — Specify independent variables, features extraction and dimension reduction, model, training and evaluation metrics.
